# Beef-Tea and How to Make It

**Published:** 1889-09-07

**Authors:** 


### Within the Wards.

### BEEF-TEA AND HOW TO MAKE IT.

In the sick-room beef-tea plays a part second to none of the foods or physics which are needed during a prolonged illness. It is, generally speaking, easily "made" or "marred." Perfect beef-tea is one of the most palatable foods a sick person can be provided with. At the opposite end of the scale is a liquid which no pig of moderately-instructed taste would consider anything but "wash." At the recent meeting of the British Medical Association, held at Leeds, Dr. Charles Moore Jessop, deputy surgeon-general, read a paper on the "Physiological Value of Meat Foods for Invalids, and Waste in Methods of Preparation." With a title so portentously long something very much above the ordinary might have been expected in the paper. It is, however, on the whole a little disappointing, though a good many hints of genuine worth may be extracted from it by careful reading. "Forty years ago," says Dr. Moore Jessop, "patients were deluged with meat washings under the belief that they were being nourished. As they did not improve, brandy was adopted; so, between brandy and meat washings, recovery was delayed." No doubt if a patient's diet consisted entirely of "brandy and beef washings" recovery would be very considerably delayed, if, indeed, in some cases it was not postponed altogether.

Medical men, says Dr. Moore Jessop, know perfectly well what good beef-tea is, but patients complain that they do not give easy directions for its preparation. That, however, he thinks, is sometimes the fault of the attendant, who assures the physician that she "knows all about it." The reader of Dr. Moore Jessop's paper may complain, and not without reason, that he, too, fails in making some of his directions "easy." Any quantity of learned matter, culled with much diligence from books and medical newspapers, is "to the fore;" but simple directions, available for the ordinary reader, are reduced to a minimum. Perhaps the writer of the paper may argue that he did not write for the ordinary reader. It is not quite impossible, however, that among his hundreds of medical hearers there may have been some whose knowledge of the best method of making beef-tea was a little nebulous, and therefore, even for them, a few plain and simple directions would have been by no means out of place. We confess to a liking for simplicity in all kinds of writing. A Scotch minister used to preach such peculiar sermons that nobody in the world could understand them. Many of his hearers thought the preacher must be too deeply learned for their comprehension. A shrewd old woman, however, expressed the opinion that the Rev. Dr. —— was "no sae deep, but just drumly." Many writers and speakers who flatter themselves that they are very deep are "just drumly." However, Dr. Jessop protests against "wash" instead of beef-tea, and therein he hits a very important nail exactly on the head.

What then is "wash" in this connection? It is "clear" beef-tea. "Nurses, cooks, and patients, all clamour for clear beef tea," says the doctor. "Clear beef-tea is of no use to anyone as food. It is true it is just what the patients like, and what they can be got to take; but what is proper for sustenance is lost sight of. However well (such) beef-tea may be made *the most important part is left at the bottom of the cup*." That part is the grounds, which are often thought, both by patient and nurse, to be "of no use." Ordinary meat for beef-tea consists of 78 parts of water and 22 of solids in the 100. The solids are for the most part albuminoids, and when introduced into the stomach in a fit condition are very easy of assimilation. "The duty of this nitrogenous substance is to develop and renovate the tissues, assist in the formation of secretions, and produce force." Hence the solid parts of the beef should be so prepared as to be capable of mixing well with the fluid parts, and thus a beef-tea will be produced which will not only be a pleasant drink and an agreeable stimulant, but a nutritious food.

This contention that purely fluid beef-tea is of little use as nourishment, Dr. Jessop supports by reference to very high authorities. The great Liebig himself is made to bear testimony on the point, and the *Lancet* is quoted to the effect that "Dogs fed exclusively on Extractum Carnis die sooner than those not fed at all," a fact which "seems to be due to the deleterious influence of the potash salts contained in the extract. For though these are indispensable in the economy, yet a larger dose of them is injurious in the absence of food whose metabolism it is their office to direct." . . . . "A patient may be swallowing several ounces of extract daily, and yet be actually starving. He may feel better for it (at the time), but his strength will not return unless he can swallow something else as well. We have eaten half a pot of the stuff, and remained as hungry as ever, the only effect being to produce thirst, to heighten the bodily temperature, and to increase the nitrogenous waste in the urine." What, then, is the use of the extracts of beef and the beef-teas that are made without the solid parts of the beef being retained? They are, according to Dr. Moore Jessop, "good stimulants," and so serve a useful purpose in the sick-room; they are also of service in bringing up the flavour of soups. In large doses, unaccompanied by a suitable quantity of food, they may very easily do much more harm than good.

Dr. Moore Jessop says that, in making fluid meat food, there should be neither refuse nor remainder. The whole of the lean meat employed should be used up. If 100 pints of fluid meat food are required, he would advise that they be made in the following way; 25lbs. of good beef are to be taken, and the meat, being first well chopped or minced, is to be placed in a digester with 100 pints of water, and boiled for three hours, being *frequently stirred and rubbed about with a wooden masher*. At the end of that time the fibre will be cooked. The mass should then be passed through a colander to ensure that every individual fibre is reduced to a size suitable for fluid food. If the process has been properly managed there will be no remainder. The food can then be seasoned with salt, onions, or other appetiser. The addition of "stock," which the doctor contemptuously describes as "stuff," is not required. The method of Dr. Jessop has at least the merit of simplicity. We have not ourselves tried it, but it is undoubtedly well worth an experiment. Of course, it can be tried with one pound of meat and four pints of water, as well as with twenty-five pounds and 100 pints of water. With Dr. Jessop's contention as to the superior nutrient value of beef-tea in which the solid fibre is suspended we entirely agree.

